# Charting the Information and Misinformation Landscape to Characterize Misinfodemics on Social Media: COVID-19 Infodemiology Study at a Planetary Scale

**DOI:** 10.2196/32378

**Published:** 2022-02-08

**Authors:** Emily Chen, Julie Jiang, Ho-Chun Herbert Chang, Goran Muric, Emilio Ferrara

**Affiliations:** 1 Information Sciences Institute University of Southern California Marina del Rey, CA United States; 2 Department of Computer Science University of Southern California Los Angeles, CA United States; 3 Annenberg School of Communication University of Southern California Los Angeles, CA United States; 4 Keck School of Medicine University of Southern California Los Angeles, CA United States

**Keywords:** social media, social networks, Twitter, COVID-19, infodemics, misinfodemics, infodemiology, misinformation

## Abstract

**Background:**

The novel coronavirus, also known as SARS-CoV-2, has come to define much of our lives since the beginning of 2020. During this time, countries around the world imposed lockdowns and social distancing measures. The physical movements of people ground to a halt, while their online interactions increased as they turned to engaging with each other virtually. As the means of communication shifted online, information consumption also shifted online. Governing authorities and health agencies have intentionally shifted their focus to use social media and online platforms to spread factual and timely information. However, this has also opened the gate for misinformation, contributing to and accelerating the phenomenon of misinfodemics.

**Objective:**

We carried out an analysis of Twitter discourse on over 1 billion tweets related to COVID-19 over a year to identify and investigate prevalent misinformation narratives and trends. We also aimed to describe the Twitter audience that is more susceptible to health-related misinformation and the network mechanisms driving misinfodemics.

**Methods:**

We leveraged a data set that we collected and made public, which contained over 1 billion tweets related to COVID-19 between January 2020 and April 2021. We created a subset of this larger data set by isolating tweets that included URLs with domains that had been identified by Media Bias/Fact Check as being prone to questionable and misinformation content. By leveraging clustering and topic modeling techniques, we identified major narratives, including health misinformation and conspiracies, which were present within this subset of tweets.

**Results:**

Our focus was on a subset of 12,689,165 tweets that we determined were representative of COVID-19 misinformation narratives in our full data set. When analyzing tweets that shared content from domains known to be questionable or that promoted misinformation, we found that a few key misinformation narratives emerged about hydroxychloroquine and alternative medicines, US officials and governing agencies, and COVID-19 prevention measures. We further analyzed the misinformation retweet network and found that users who shared both questionable and conspiracy-related content were clustered more closely in the network than others, supporting the hypothesis that echo chambers can contribute to the spread of health misinfodemics.

**Conclusions:**

We presented a summary and analysis of the major misinformation discourse surrounding COVID-19 and those who promoted and engaged with it. While misinformation is not limited to social media platforms, we hope that our insights, particularly pertaining to health-related emergencies, will help pave the way for computational infodemiology to inform health surveillance and interventions.

## Introduction

As COVID-19 forced more of the world to undergo lockdowns and to adopt physical distancing, the public sought refuge and community support online to replace the interactions that were no longer possible in person. Social media platforms soon became a means for messaging involving the COVID-19 pandemic, with policy makers and medical experts taking to social media to reach the public, and the public using these platforms as forums for debate and information exchange.

Twitter remains one of the main platforms used as a vehicle for communication in the COVID-19 era. This and other similar platforms, however, enabled false or misleading information with the potential to cause harm to public health to take root. The increasing reliance on platforms as a means for communication during COVID-19 underscored the importance of *infodemiology*, which is the study of the spread of “health information and misinformation” on online platforms [1,2], and brought the concept of *infodemics*, defined as the epidemic-like spread of information, to the public eye [3]. While the intensity of its effects varies based on country and culture, infodemics was and continues to be a salient issue in COVID-19 discourse [4,5]. Misinformation, particularly during a pandemic, can dissuade some individuals from readily adopting health practices that would contribute to curbing the spread of the disease [6]. Efforts are being made to combat misinformation, including identifying intervention points in social networks to mitigate misinformation [7], teaching the community how to identify misinformation [6], rating source reliability [8], and using both crowdsourced and official fact checkers to identify misinformation [9,10]. Social media platforms have also begun adding notifications to remind users to be cautious when reading certain information [11].

**Figure 1:**
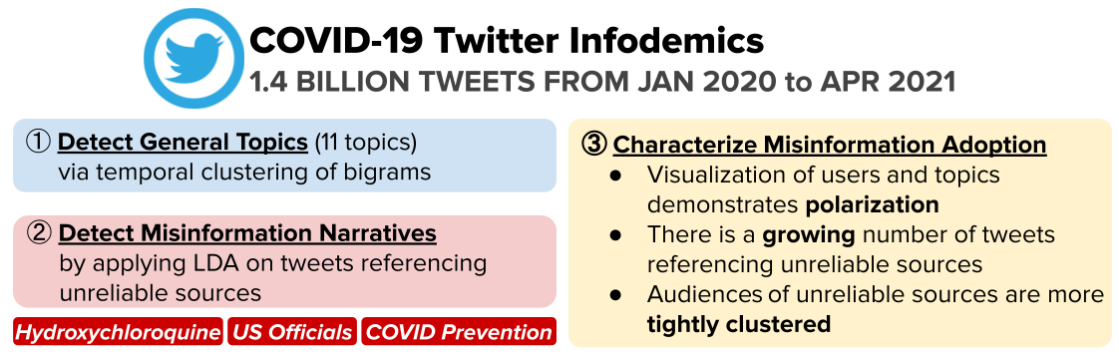
Overall roadmap of this paper. LDA: latent Dirichlet allocation.

In this paper, we take a deeper look into both the general COVID-19 conversation and the misinformation narratives on Twitter between January 2020 and April 2021 (Figure 1). The contributions we make in this paper are as follows: (1) We identified 11 major topics of general discussion present throughout our overarching data set, which are temporally in line with the progression of current events; (2) We detected 3 prominent misinformation narratives (namely, hydroxychloroquine and alternative medicines, US officials and governing agencies, and COVID-19 prevention efforts); (3) We found that there are distinct political echo chambers and that a user’s political alignment is linked to the misinformation narratives the user engages with; and (4) We took a closer look at the types of misinformation domains that are shared and found that the consumption of conspiratorial and questionable content is on the rise. Users who share unreliable health-related content also tend to be in more tightly connected communities compared with the average Twitter user.

## Methods

### Data

We began collecting and curating a COVID-19 Twitter data set right at the beginning of the pandemic, in January 2020, to continuously track, in real time, public discourse about the coronavirus pandemic. We have made the data set publicly accessible to the wider research community [12]. This study uses publicly available data, and the data collection and analysis are approved by the University of Southern California Institutional Review Board (protocols UP-17-00610 and UP-21-00005).

Our complete data set, as of this writing (mid-July 2021), contains 1,497,893,426 tweets from January 21, 2020, through July 9, 2021 (release v2.55). While we provide a brief overview of our data set here, a full description of our data set can be found elsewhere [12]. We leveraged release v2.45 for this paper, which contains 1,443,871,621 tweets from January 21, 2020, through April 30, 2021. All our tweets were collected in real time using Twitter’s streaming application programming interface (API), which gave us access to a 1% stream of tweets [13]. We leveraged a manually curated list of keywords to filter for tweets that contained content related to the COVID-19 pandemic and surrounding issues. We list a sample of the keywords we tracked in Table 1. The full list of up-to-date keywords can be found in our GitHub repository [14]. While we did our best to capture as much discourse as we could in our collection, a limitation of our data set is that our keywords were all in English and were manually selected for tracking. This may have influenced the collected tweets and our subsequent observations. A language breakdown for the tweets found in release v2.45 can be found in Table 2.

**Table 1:** A sample of keywords that were tracked during this release (v2.45; May 3, 2021).

Keyword^a^	Tracked since
Coronavirus	January 28, 2020
CDC	January 28, 2020
Wuhanlockdown	January 28, 2020
Kungflu	January 28, 2020
corona virus	March 2, 2020
covid	March 6, 2020
covid19	March 6, 2020
sars-cov-2	March 6, 2020
COVID–19	March 8, 2020
coronapocalypse	March 13, 2020
SocialDistancing	March 13, 2020
shelteringinplace	March 18, 2020
flatten the curve	March 18, 2020

^a^We do not need to track every permutation of a keyword. As of this writing, Twitter returns all tweets that contain the keyword as a substring, and it is case insensitive.

**Table 2:** The top 10 languages and their prevalence in all tweets collected in this release (v2.45; May 3, 2021).

Language^a^	ISO^b^	Tweets (N=1,443,871,621), n (%)
English	en	928,225,493 (64.29)
Spanish	es	186,880,167 (12.94)
Portuguese	pt	62,398,113 (4.32)
French	fr	44,097,563 (3.05)
Undefined	und	41,140,188 (2.85)
Indonesian	in	35,683,876 (2.47)
German	de	25,970,256 (1.80)
Japanese	ja	16,865,989 (1.17)
Italian	it	15,697,293 (1.09)
Turkish	tr	14,931,506 (1.03)

^a^The language tags are automatically detected by Twitter and returned in the tweet metadata.

^b^ISO: International Organization for Standardization.

#### Identifying Discussion Topics

To understand the general COVID-19–related topics that were discussed on Twitter, we identified the bigrams (ie, consecutive word pairs) used in our data set and clustered bigrams that share similar temporal usage characteristics.

##### Bigrams

To retrieve bigrams, we first tokenized the tweets, lowercased all tokens, and removed stop words and select punctuations (including hash signs used for hashtags in Twitter). For example, the (fictitious) tweet “Thousands of new #covid cases reported in Los Angeles County!!” reduces to the sequence of tokens “thousands new covid cases reported los angeles county;” all bigrams would be extracted, such as “thousands new,” “new covid,” “covid cases,” “cases reported,” etc. To avoid sparsity of data and to reduce computational costs, we focused on only the 50,000 most frequent bigrams that appeared in this data set. We replicated this step with 10,000 and 100,000 bigrams and found the results to be consistent. We built a time-series vector for each bigram to characterize its popularity over time. This time series was built by counting the number of times each selected bigram was used on a weekly basis and normalizing that count by the total number of bigrams used that week.

##### Temporal Clustering

**Figure 2:**
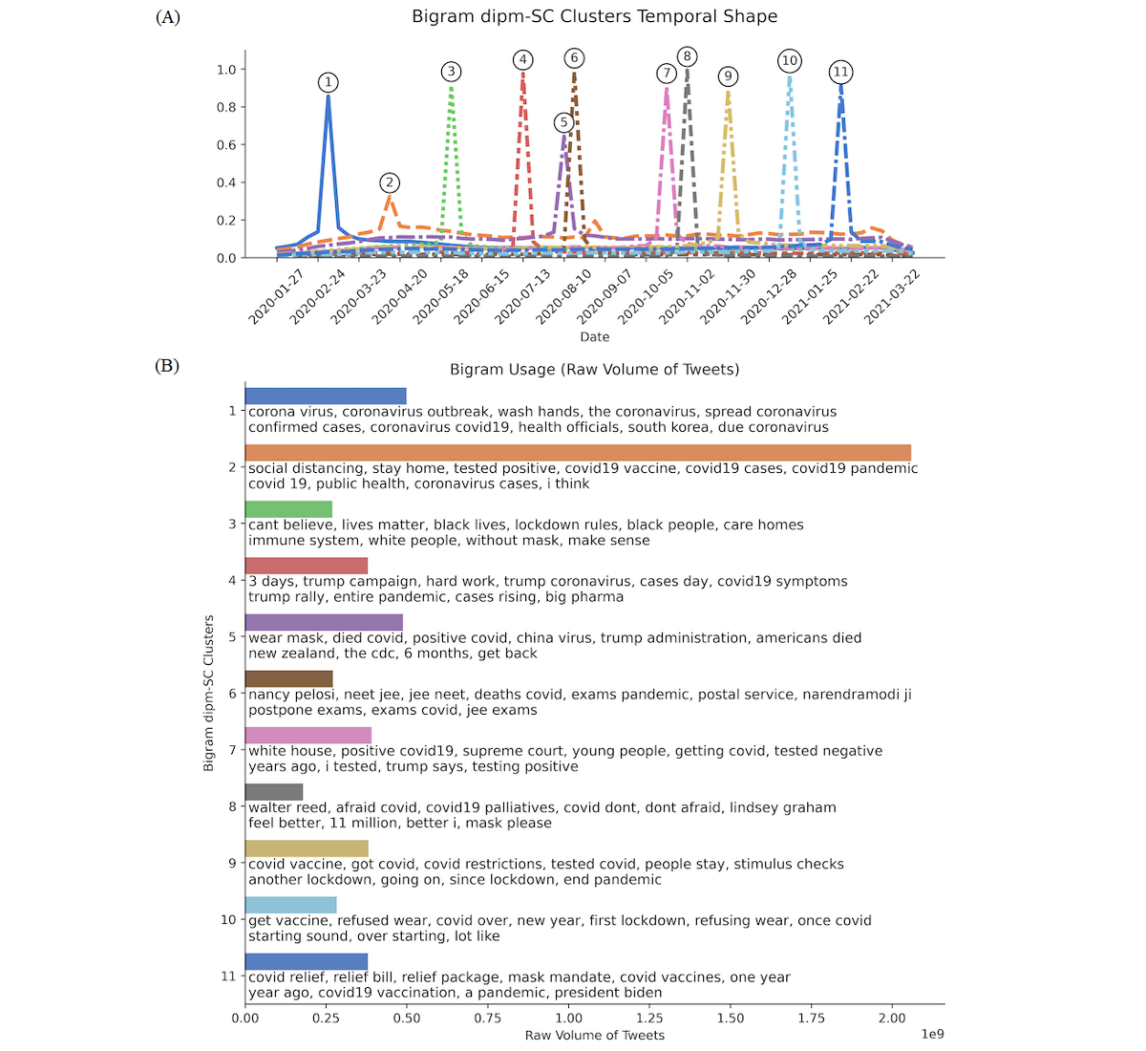
(A) Detected shapes of identified clusters, ordered by when each cluster peaked in popularity. Each line indicates the respective cluster’s popularity over time. (B) The top 10 most used bigrams associated with each cluster and bar chart showing their total usage in terms of raw volume of tweets. The 11 clusters were (1) general coronavirus concerns, (2) public health measures, (3) Black Lives Matter, (4) Trump rallies, (5) 6 months after the first COVID-19 case, (6) Indian national exams, (7) the second COVID-19 wave, (8) Trump tests positive, (9) vaccine development, (10) vaccine rollout, and (11) COVID relief bill.

With the normalized bigram usage counts, we used *dipm-SC* [15], a shape-based time-series clustering algorithm that we designed specifically for social media data. The algorithm finds *K* clusters of bigrams that exhibit similar temporal behaviors, within a certain prespecified time window *W*. We set the window to *W*=21 days to detect topics that had been trending for at the most 3 weeks, automatically filtering out general trending topics that had a tendency to continuously dominate the discussion over time (eg, bigrams like “covid 19” or “corona virus”). The results were consistent with similar assignments of *W*. We also explored various settings of *K*, the number of clusters, ranging from 5 to 15. While results were robust with similar assignments of *K*, we found that *K*=11 produced the optimal number of clusters in terms of the coherency of extracted topics and the amount of temporal overlap observed in the detected temporal shapes (eg, Figure 2A) via manual inspection.

##### Topic Clustering

Latent Dirichlet allocation (LDA) [16] is a popular topic modeling approach, which finds *N* latent topics in a group of documents (in our case tweets). The number of clusters (or topics) that yields the largest *coherence value* is determined to be the optimal *N* value [16]. We again tokenized, lowercased, and removed all stop words and select punctuations from the tweets, and used LDA to cluster tweets by general topic. We found that *N*=4 yielded the largest coherence value.

#### Misinformation Subset

From our broader COVID-19 data set, we wanted to understand the kinds of narratives and discourse that promoted questionable content and misinformation. We created a subset of our data set for published tweets that contain a URL belonging to a domain that has been determined to be prone to publish questionable or conspiracy-pseudoscience–related content according to the third-party service Media Bias/Fact Check (MBFC) [17]. We used this as a proxy to identify users who have engaged with misinformation. This resulted in a COVID-19 misinformation data subset totaling 12,689,165 tweets.

### Identifying Conspiratorial, Questionable, and Random Sources

To identify conspiratorial and questionable tweets, we used the following 2 lists compiled by MBFC: *conspiracy-pseudoscience sources* and *questionable sources*. MBFC is “an independent website that rates the bias, factual accuracy, and credibility of media sources” [17]. MBFC classifies domains as *conspiracy-pseudoscience* if the domain “*may* publish unverifiable information that is *not always* supported by evidence. These sources may be untrustworthy for credible or verifiable information” [17]. For the sake of brevity, we also refer to these conspiracy-pseudoscience domains as simply *conspiracy* or *conspiratorial* domains. MBFC states that questionable sources are domains that “exhibit one or more of the following: extreme bias, consistent promotion of propaganda/conspiracies, poor or no sourcing to credible information, a complete lack of transparency and/or is fake news. Fake news is the deliberate attempt to publish hoaxes and/or disinformation for the purpose of profit or influence” [17].

We also obtained a set of randomly selected sources by taking a random sample from the set of media sources that appeared in the full data set. We called this set of sources “*random* sources.” The set of *random* sources has the same number of elements (URLs) as *conspiratorial* and *questionable* sources. The *random* sources served as a baseline for comparison with *conspiratorial* and *questionable* sources.

### Identifying a Source’s Political Bias

MBFC also classifies media domains by their political affiliations, with the following 5 political affiliation categories: left bias, left-center bias, least biased, right-center bias, and right bias. We used their lists of domains to identify tweets with a particular political affiliation. Left and right bias sources are “moderately to strongly biased,” may be untrustworthy, and can “publish misleading reports and omit reporting of information that may damage [their] cause” [17]. Left-center and right-center bias sources have “slight to moderate” bias and are “generally trustworthy for information but may require further investigation” [17]. MBFC goes on to describe sources tagged as least biased as sources with “minimal bias,” “factual and usually sourced,” and “the most credible media sources” [17].

### Classifying a User’s Misinformation and Political Engagement

For every user in our misinformation subset, we tabulated the number of times they shared domains and identified the political bias of these domains. This gave us a proxy of each user’s political lean. The political lean was determined by the political lean of the majority of a user’s shared domains. In the case of a tie between 2 political biases, we randomly assigned the user a political bias. Any user who shared one or more questionable or conspiracy-pseudoscience domains (as identified by MBFC) within our data set was considered to have engaged with misinformation. This does not mean that a user in our misinformation subset exclusively or mostly shared misinformation content. We restricted our analysis to only users who had shared more than five URLs.

### User Retweet Network Misinformation Analysis

Taking advantage of the retweeting dynamics of Twitter, we constructed a network to conduct social network analysis on the users in our misinformation subset. Nodes represent users and links (or ties) represent retweets between users. If user A (retweeting) retweets user B (retweeted), then the strength of their tie increases with the frequency of retweets. To visualize this network, we adopted a force-based algorithm, Force Atlas [18], which plots nodes that share strong links close together. For the sake of clarity, the ties are not explicitly shown. There were a total of 4,164,572 users and 22,894,165 unique ties between users in our misinformation subset. We labeled the most prominent users, sorted by their highest out-degree. 

This retweet network is constructed from the tweets of users who had retweeted at least one tweet that contained a domain that MBFC had classified as a questionable or conspiracy-pseudoscience domain. This means that each link between a retweeted and retweeting user does not necessarily mean that the retweet contained a misinformation domain or that the retweeted user engaged with a misinformation domain. Thus, the entire retweet network (contained within our dataset) included users who had interacted with a misinformation domain at least once.

### Linear Regression Model Over Time

We analyzed the content coming from the following 3 groups of sources, each containing 250 URL domains: *conspiracy* sources, *questionable* sources, and *random* sources. *Conspiracy* and *questionable* sources were domains classified as such by MBFC, whereas *random* sources were chosen from a set of URLs selected at random to serve as a baseline for comparison.

To calculate the temporal trends in the amount of news coming from unreliable sources, we performed 2 multiple linear regression analyses using standard ordinary least-squares models. The first model estimated the association between the number of *conspiratorial* URLs and time, adjusting for an average number of URLs observed on a platform. The model can be represented as follows: *V_C_ ~ tβ_1_ + V_R_ β_2_*, where *V_C_* is the number of *conspiratorial* URLs shared, *t* is time measured in days, and *V_R_* is the number of *random* URLs shared on Twitter. The second model estimated the association between the number of *questionable* URLs and time, adjusting for an average number of URLs on a platform. Similarly, it can be represented as follows: *V_Q_ ~ tβ_1_ + V_R_ β_2_,* where *V_Q_* is the number of *questionable* URLs shared.

### Domain Sharing Network Analysis

To better understand the relative impact of unreliable sources, we looked at their respective audiences and the communities that formed around sharing these unreliable sources. It is important to quantify the community structure and relationships between the consumers of certain kinds of information, as the strength of these communities can be indicative of the potential of an idea within the community to grow and become dominant over time. According to organizational theory, interpersonal networks that exhibit densely configured ties have a greater likelihood of attaining their goals and retaining the network structure (committed to staying together). Networks of strong ties are also significantly more robust with respect to the connectivity and small world property of social networks [19,20].

To quantify the relative strength of a connection between information sources that spread unreliable information about COVID-19, we constructed 3 networks of the following group of domains as defined earlier: *conspiracy*, *questionable,* and *random* sources. The nodes in the network represent the domains, and a link was drawn between 2 domains if a user shared content from both domains. The weight of a link was set to the number of users who shared both domains. To quantify the density of connections in these networks, we calculated the average clustering coefficient [21] and the average link weight for each respective audience network.

## Results

### Clusters of Major Discussion Topics About COVID-19 on Twitter

We used a clustering strategy based on *dipm-SC* [15], described in the Methods section, to identify topics that exhibit similar temporal behaviors and group them into distinct clusters. The detected clusters are visualized in Figure 2. We found that all clusters exhibited distinct peaks, suggesting minimal overlap between distinct clusters and hence robust and reliable clustering results. We now briefly describe the key topics that were detected in the 11 clusters we identified.

#### General Coronavirus Concerns

This concerns general coronavirus-related tweets, including reminders to “wash hands,” which was the first and most repeated advice to safeguard against the virus. It peaked in popularity early in the outbreak, in January and February 2020. It gradually declined in popularity until June 2020, from which point on it sustained its popularity consistently by accounting for around 10% of all tweets. This topic’s popularity trajectory tracks well with the initial phase of the COVID-19 outbreak unfolding worldwide.

#### Public Health Measures

Messages promoting public health measures, such as “social distancing” and to “stay home,” have been popular during COVID-19. This kind of messaging peaked in popularity during March and April 2020, after the lockdowns were imposed, and commanded attention throughout the rest of the study period. While this cluster had the shortest peak in terms of temporal shapes, we noticed that it was overwhelmingly the single most popular topic of all time points (Figure 2B). This contrast is due to the fact that this trending topic is relatively steady overtime rather than bursty during a short timeframe, like the other clusters. The high level of total activity indicates the high level of attention that the Twitter audience paid to public safety measures.

#### Black Lives Matter

The killings of George Floyd, Breonna Taylor, and others sparked national outrage [22]. This topic was brought up along with COVID-19 in late May through early June due to concerns that public protests would increase case counts. The protests were later found to have had no significant impact on the number of COVID-19 cases [23].

#### Trump Rallies

In June, former President Trump resumed his in-person rallies for his 2020 presidential re-election campaign. Rallies had been halted due to widespread coronavirus concerns over in-person gatherings [24].

#### Six Months After the First COVID-19 Case

Six months after the first COVID-19 case was reported, people were still battling the pandemic and isolating at home, unable to resume normal activities. The topic also includes the Trump administration’s use of the anti-Asian term “China virus.”

#### Indian National Exams

This temporal cluster of bigrams is primarily concerned with India’s NEET and JEE national exams, which had been postponed twice due to COVID-19. This became controversial when the exams were scheduled for September 2020 during a time when cases in India were steadily rising [25]. This topic anticipates, by several months, the outbreaks associated with the Delta variant in India that began in December 2020 [26].

#### The Second COVID-19 Wave

The United States braced itself for another wave of COVID-19 cases in September 2020 [27], with major concerns for the younger population.

#### Trump Tests Positive

On October 2, 2020, the White House announced that former President Trump tested positive for the coronavirus; soon after, Trump was transported to Walter Reed Medical Center [28].

#### Vaccine Development

By November 2020, both Pfizer and Moderna published promising results regarding their vaccines [29]. Shortly thereafter, both vaccines were approved for emergency use by the United States Food and Drug Administration (FDA) [30].

#### Vaccine Rollout

In the final weeks of 2020, vaccine administration began rolling out in the United States and in many other parts of the world [29,30].

#### COVID Relief Bill

After more than a year since the first case of COVID-19 was reported, many parts of the world continued to operate under mask and social distancing mandates. The vaccine rollout promised to facilitate a long-anticipated return to normalcy. The 2021 COVID-19 stimulus package, or American Rescue Plan Act, was eventually passed and was signed into law in March, which amounts to US $1.9 trillion [31].

### COVID-19 Misinformation Narratives

We then turned to investigating misinformation and questionable narratives that spread in the context of COVID-19. We used our misinformation data subset, which contains tweets with URLs whose domains were deemed to be from a conspiracy-pseudoscience or questionable source according to MBFC, and leveraged both *dipm-SC* [15] and LDA [16] to cluster tweets by general topic. From the topics found in both clustering methods, we identified the following 3 major misinformation narratives that encapsulate the tweets that spread questionable media content on Twitter: (1) hydroxychloroquine and alternative medicines, (2) US officials and governing agencies, and (3) COVID-19 prevention interventions.

**Figure 3:**
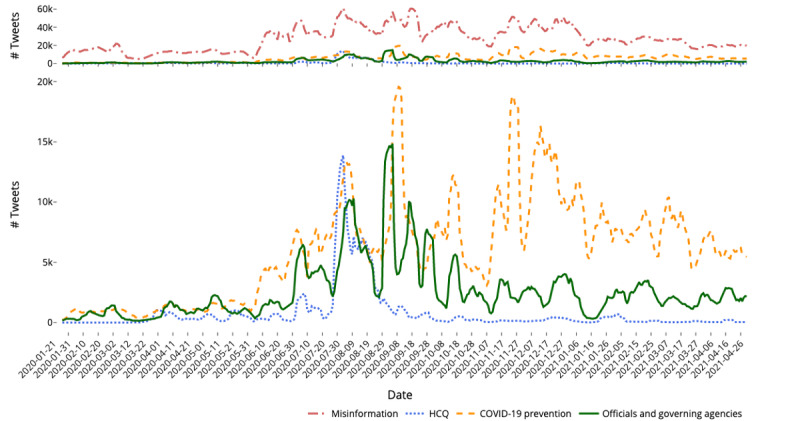
Visualization of the 7-day moving average of the volume of tweets that have tweeted a URL from a domain that has been identified as having spread conspiracy-pseudoscience or questionable content according to Media Bias/Fact Check. We identify 3 major narratives and plot the volume of tweets over time that mention keywords related to each of the narratives (hydroxychloroquine [HCQ], US officials and governing agencies, and COVID-19 prevention) in the bottom figure. The top figure plots the same narratives but also includes the total volume of tweets that shared a conspiracy-pseudoscience or questionable domain (which we generalize as misinformation).

For each narrative of interest, we filtered our misinformation data set based on several defining keywords (Table 3). We identified the keywords in Table 3 by first isolating the most used keywords and bigrams in each narrative’s cluster, and then manually selecting neutral keywords most reflective of the 3 narratives. This enabled us to isolate subsets of tweets that specifically mentioned keywords related to each misinformation narrative. We then plotted the volume of tweets from each narrative over time (Figure 3) to understand temporal trends in each narrative. We found that a constant flow of misinformation exists, despite Twitter’s efforts to mitigate its spread. However, when we isolated tweets by narratives, we saw that each narrative experiences differing levels of engagement over time. Most of these spikes are driven by active retweeting of viral posts and/or articles that are sometimes related to real-time events. For each narrative, we also found the top hashtags that were used and grouped them into their relevant categories. We did a manual inspection of the tweets during these peaks and describe a few of the prominent topics that drove the volume surges in each narrative as seen in Figure 3.

**Table 3:** Tweets isolated from our misinformation data set that are related to each topic by filtering specific topic-related keywords (N=12,689,165).

Topic^a^	Keywords	Total number of tweets
Hydroxychloroquine and alternative medicines	hcq, hydroxychloroquine	368,883
US officials and governing agencies	fauci, brix, cdc	1,205,824
COVID-19 prevention	mask, vaccine, social distanc*, test	2,804,985

^a^Note that a tweet can fall under multiple topics and count toward the narrative’s total number of tweets.

#### Hydroxychloroquine

Hydroxychloroquine was, at the beginning of the pandemic, considered to be a potential treatment for COVID-19. However, while the US FDA had issued an emergency use authorization for the drug and the World Health Organization (WHO) had considered hydroxychloroquine in clinical trials, the drug had not been proven to be effective against the novel coronavirus [32,33]. As it became clear that hydroxychloroquine was not an effective treatment, the US FDA withdrew the emergency use authorization in June 2020 [32,33] and the WHO removed it from its trials in July 2020 [34]. Despite the evidence of inefficacy brought by clinical testing, hydroxychloroquine remained a fixture to many as an alleged cure for the coronavirus, and henceforth, it is considered medical misinformation. The top hashtags used in this narrative can be found in Textbox 1.

**The top 20 hashtags from the misinformation data set related to hydroxychloroquine and alternative medicines (classified into 5 general topics).**

**Hydroxychloroquine-related**

hydroxychloroquine, hcq, hcqworks, hydroxychloroquineworks, and earlytreatmentworks

**General coronavirus**

covid19, coronavirus, and covid

**Fauci**

arrestfauci, fauci, firefauci, politicalcoup, and liberalfascism

**Politics**

kag, tds, twgrp, and faucifraud

**Misinformation**

ccpvirus, chinavirus, and scamdemic

##### Period From July 30, 2020, to August 14, 2020

Upon a manual inspection of the most prevalent content, we found that many users on Twitter were still circulating early and preliminary studies that suggested that hydroxychloroquine might be a candidate for treating COVID-19. Many of these users also blamed Dr Anthony Fauci and other medical authorities for ignoring the alleged “evidence” that hydroxychloroquine was effective. These users also cited the Ohio Department of Health’s prohibition on the use of hydroxychloroquine that was announced but rescinded before its July 30, 2021, effective date [35,36]. Finally, Twitter and other social media platforms began removing viral videos that featured Dr Stella Immanuel promoting unproven and unsubstantiated claims that hydroxychloroquine was an effective treatment for COVID-19 [37]. This resulted in users who engaged in hydroxychloroquine misinformation during this time claiming that Twitter was attempting to violate their freedom of speech.

#### US Officials and Governing Agencies

Perhaps unsurprisingly, US officials and governing authorities were also a target for misinformation on online platforms such as Twitter. Given that our data set was curated with English keywords, there was a higher concentration of discourse surrounding events occurring in primarily English-speaking countries. In our prior work, we also found that a large percentage of Twitter users were located in the United States [38]. Thus, the major misinformation narratives surrounding authorities centered around US officials and authority figures. The top hashtags used in this narrative can be found in Textbox 2.

**The top 20 hashtags from the misinformation data set related to US officials and governing agencies (classified into 4 general topics).**

**General coronavirus**

coronavirus, covid19, cdc, covid, vaccine, and vaccines

**Fauci**

fauci, firefauci, faucithefraud, arrestfauci, and anthonyfauci

**Misinformation**

qanon2018, qanon2020, thedefender, ccpvirus, and chinesecoronavirus

**Miscellaneous**

trump, china, un, and who

##### Period From July 4, 2020, to July 8, 2020

Users cited a report that the Centers for Disease Control and Prevention (CDC) was overcounting COVID-19 cases and used this to claim that the CDC was purposefully trying to force Americans to remain under lockdowns throughout the summer [39,40].

##### Period From August 4, 2020, to August 10, 2020

Reports from the far-right news outlet The Gateway Pundit surfaced claims from Robert F Kennedy Jr, an antivaxxer who was banned from Instagram in February 2021 for spreading misinformation [41]. He claimed that Dr Anthony Fauci would be heavily profiting off the success of vaccines, falsely stating that Fauci was a partial owner of a COVID-19 vaccine patent [42]. There was also another report from The Gateway Pundit that disparaged US government medical authorities for downplaying the benefits of hydroxychloroquine and ignoring lower mortality rates in countries that used hydroxychloroquine as a treatment [43].

##### Period From August 30, 2020, to September 4, 2020

The Gateway Pundit published a report claiming that only 9210 Americans had died specifically from COVID-19, while all other deaths were related to other illnesses [44]. They then used this as grounds to push the narrative that the CDC was overreacting to and exaggerating the effects and impact of COVID-19.

##### Period From September 15, 2020, to September 19, 2020

Former President Donald Trump issued an order for agencies to stop racial sensitivity training [45]. The Gateway Pundit published an article claiming that the CDC was disregarding Trump’s orders [46].

##### Period From September 26, 2020, to October 2, 2020

The CDC posted and then retracted a post on the airborne transmission of COVID-19 [47,48]. In reaction to the retraction, users accused the CDC of lying and intentionally misleading the public.

##### Period From October 13, 2020, to October 19, 2020

The CDC released a report that surveyed a small group of individuals who had contracted COVID-19. One of the questions posed to the participants was regarding their mask usage, and over 70% of the COVID-19 patients reported using a mask [49]. Users on Twitter used this information to bolster their belief that masks are not effective. This claim has been fact checked and debunked, showing that these users disregarded the context and other findings that these numbers were presented with [50,51].

#### COVID-19 Prevention

The last major narrative we identified in our misinformation data set focuses on COVID-19 prevention mechanisms. This includes testing, vaccines, masking, and social distancing. Many of the suggested and proven COVID-19 prevention strategies have been and continue to be at the center of much controversy, and as a result, are subject to much misinformation. The top hashtags used in this narrative can be found in Textbox 3.

**The top 20 hashtags from the misinformation data set related to COVID-19 prevention (classified into 4 general topics).**

**General coronavirus**

covid19, covid, cdc, coronavirus, covid—19, covid 19, and fda

**Prevention mechanisms**

pfizer, moderna, vaccine, vaccines, masks, lockdown, and covidvaccine

**Misinformation**

ccpvirus, billgates, and thedefender

**Miscellaneous**

unmaskamerica, hankaaron, and science

##### Period From August 2, 2020, to August 9, 2020

The Gateway Pundit interviewed Robert F Kennedy Jr, who claimed that Dr Fauci would “make millions” from vaccine developments. This is the same story that drove a peak of activity surrounding US officials and authorities (see the time frame August 4, 2020, to August 10, 2020, in the US Officials and Governing Agencies section). During this time, Ohio governor Michael DeWine tested positive with an antigen test (also referred to as a rapid test) when being screened for a White House event with former President Trump. DeWine later tested negative after taking the more accurate polymerase chain reaction test [52,53]. This discrepancy in test results, despite the known difference in accuracy, caused users on Twitter to question the necessity and effectiveness of testing.

##### Period From September 4, 2020, to September 13, 2020

The Bill and Melinda Gates Foundation has invested heavily into developing vaccines for diseases such as Polio [54]. *Zerohedge*, a far-right news blog, published a post about the United Nations reporting a new vaccine-related polio outbreak in areas of Africa, specifically identifying the vaccine as a “Gates-Funded” vaccine [55,56]. This caused conspiracy theorists who were circulating this misinformation to blame Bill Gates for supposedly “funding” polio and for benefiting from it [57,58]. The same *Zerohedge* article then used this as evidence to try to bring the efficacy and safety of COVID-19 vaccines into doubt [55].

##### Period From October 10, 2020, to October 20, 2020

Former President Trump tested positive for the novel coronavirus on October 2, 2020, and tested negative on October 12, 2020 [28,59]. The Gateway Pundit released an article attacking the efficacy and need for masks to prevent COVID-19, dismissing the CDC recommendation to wear masks [60]. The article questions the credibility of the CDC due to its initial recommendation to not wear masks and its subsequent recommendation for all to engage in mask wearing [60]. The initial policy was partially rooted in wanting to preserve the then-scarce personal protective equipment for hospital workers and those on the front line [61].

##### Period From November 13, 2020, to November 29, 2020

A post by a former Pfizer employee, Michael Yeadon, claimed that the pandemic was over in the United Kingdom and that a vaccine was not needed for COVID-19 to be overcome [62]. While this claim was debunked and marked false by news and social media platforms [63], users online capitalized on Yeadon’s past association with Pfizer, one of the producers of the COVID-19 vaccine. They cited this as validation of their belief that the pandemic was a “scam” and that vaccines are not necessary. During this time, it was also revealed that Maryland governor Larry Hogan had spent over US $9 million on COVID-19 tests that were discovered to be flawed. This caused Hogan to purchase replacements for US $2.5 million using state funds, while not disclosing these flaws [64]. *Breitbart*, a far-right news platform, criticized Hogan on this, labeling Hogan as a Republican “anti-Trump hero” for the purchase of these tests [65], which had drawn former President Trump’s ire [66].

##### Period From December 8, 2020, to December 17, 2020

Sources, such as *NationalFile* and *DailyMail*, both of which MBFC has rated as having low credibility, claimed that the Chinese Communist Party had “infiltrated” both Pfizer and AstraZeneca and that these pharmaceutical companies had provided employment to these individuals [67]. This information was then used to discredit and cast doubt upon the vaccines that both companies were producing. A claim also stated that the pharmaceutical company GlaxoSmithKline owned both the Wuhan Institute of Virology and pharmaceutical company Pfizer. These debunked claims [68,69] were an attempt to tie the Pfizer COVID-19 vaccine development to Wuhan, where the first cases of COVID-19 were reported. Finally, there was a false claim that 87,000 nurses from the Netherlands declined the COVID-19 vaccines [70,71]. This alleged “refusal” was used to promote the narrative that many medical professionals were against vaccination and as a reason for the public to also follow suit.

### Characterizing Misinformation Adoption

After identifying and describing the misinformation narratives permeating online discourse, we looked to understand the audience that is more susceptible to misinformation and the trends within the kind of misinformation that is being consumed. In the following text, we used network science as a lens to understand the structure and characteristics of misinformation echo chambers on Twitter, and suggest this as a possible mechanism to explain the spread of misinformation in specific communities.

#### Existence of Political Echo Chambers

**Figure 4:**
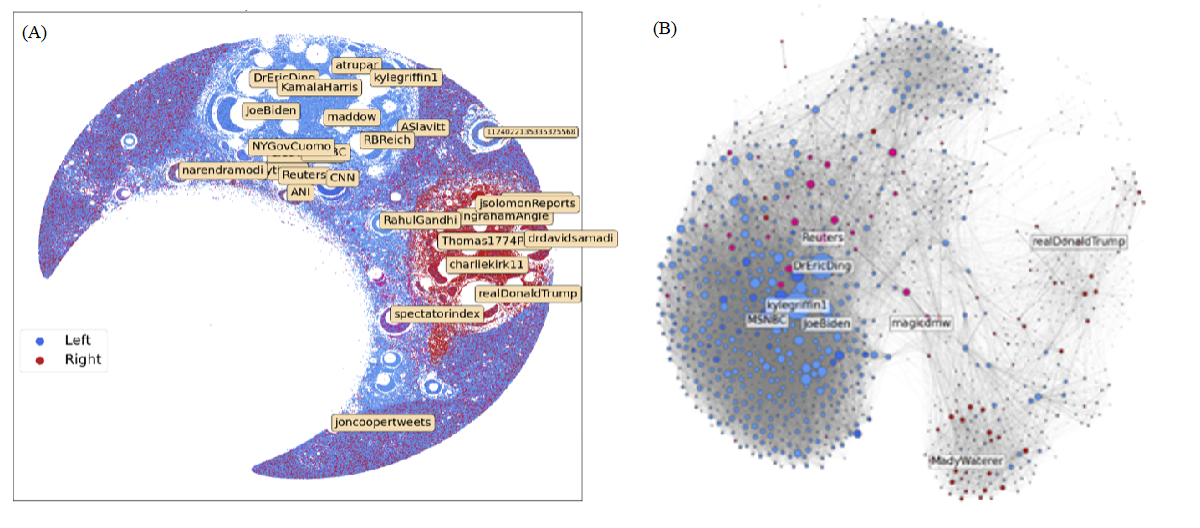
(A) The political leanings of the users within our misinformation subset. Political leanings are determined by the political affiliation (as determined by Media Bias/Fact Check) of the domains a user tweets the most. (B) The 100-core decomposition of the graph into the top 1403 accounts.

Figure 4 shows the retweet social network structure of Twitter users who engaged with at least one post containing a misinformation domain, as classified by MBFC, over the course of more than a year, which has been laid out using Force Atlas [18]. Some users, such as former President Donald Trump (*realDonaldTrump*) and President Joe Biden (*JoeBiden*), have rings of users around them, and these rings contain users that retweet almost exclusively from these prominent accounts. As a feature of the visualization, prominent users are also accompanied with “negative space” around them, which is a direct result of using the Force Atlas layout, where prominent users attract many small accounts who also repel each other. 

Figure 4 is helpful for revealing the overall structural properties of the Twittersphere and their interplay with the political orientation of users. By labeling the political diet of users based on the MBFC-classified political affiliation of the domains they share, we observed strong polarization across right- and left-leaning users. Right-leaning users (Figure 4, nodes in red) clustered around former President Donald Trump, David Samadi (physician and contributor to conservative news source Newsmax), Charlie Kirk (conservative activist), and other prominent right-leaning figures. Left-leaning users (Figure 4, nodes in blue) clustered around prominent liberal leaders, such as President Joe Biden and Vice President Kamala Harris, in addition to certain journalists and physicians. Interestingly, international media outlets, such as BNO news, SkyNews, and Spectator Index, attracted a mix of both left- and right-leaning users, suggesting that they are more impartial than US-based media outlets.

Figure 4B further breaks down the visualization through a 100-core decomposition. Here, we additionally pruned out bots by removing those who tweet frequently but are never retweeted. This showed a similar partition of the network into communities, with left-leaning users on the left and right-leaning users on the right. Among elite users, as generated by the K-core decomposition, we can see how many more left-leaning users are engaging with COVID-19 messaging.

#### Discussions of the Misinformation Narratives are Politically Fractured

**Figure 5:**
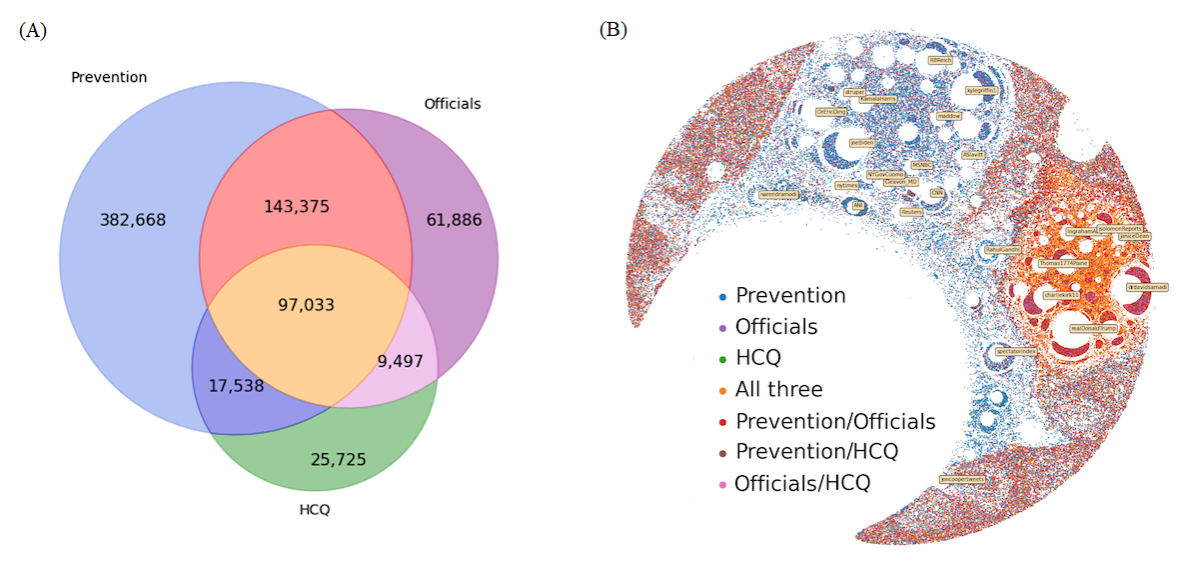
Frequency of tagged users and their overlap. (A) Their numeric overlap. (B) Their overlap on the social network visualization from Figure 4A. HCQ: hydroxychloroquine.

Given the political orientation of users and the central users for which they coalesce around, we considered how the 3 narratives from Table 3 emerge. Figure 5A shows the overlap of these topics, aggregated over all users. We observed that users engaged primarily with COVID-19 prevention discourse, followed by discussion of US officials and governing authorities, and then hydroxychloroquine and alternative medicines. Additionally, 97,033 users discussed all 3 (Figure 5A), making up 13% of the 737,722 users tagged for engaging in these 3 topics.

What is more interesting is how these topics map on the Twitter social network, as illustrated in Figure 5B. We observed COVID-19 prevention discourse throughout the graph. However, within the left-leaning cluster from Figure 4, we observed an absence of discourse about US officials and hydroxychloroquine. Users near the conservative core in Figure 4 are active in nature, and their position in the network is indicative of their higher retweeting frequency. Two types of users emerged from the right-leaning cluster. One type included users who discuss both prevention and US officials (Figure 5, red portion). They appeared concentrated around specific prominent users, such as Donald Trump and Dr Samadi (these users are labeled in Figure 4). The other type included users who engaged in discourse about all 3 narratives (Figure 5, orange portion) in tandem. These users tended to retweet a diverse number of prominent users. This not only indicates that hydroxychloroquine-related discourse is largely concentrated around right-leaning users and absent among left-leaning users, but also suggests that there exists a fracture within the right-leaning base, with some users following political content exclusively and others engaging more generally with COVID-19 discourse. Additionally, we can conclude that hydroxychloroquine is contingent on the presence of 1 of the 2 other topics (US officials and COVID-19 prevention).

### Social Media Consumption of Unreliable Sources

#### The Rise of COVID-19 Information Coming From Unreliable Sources

**Figure 6:**
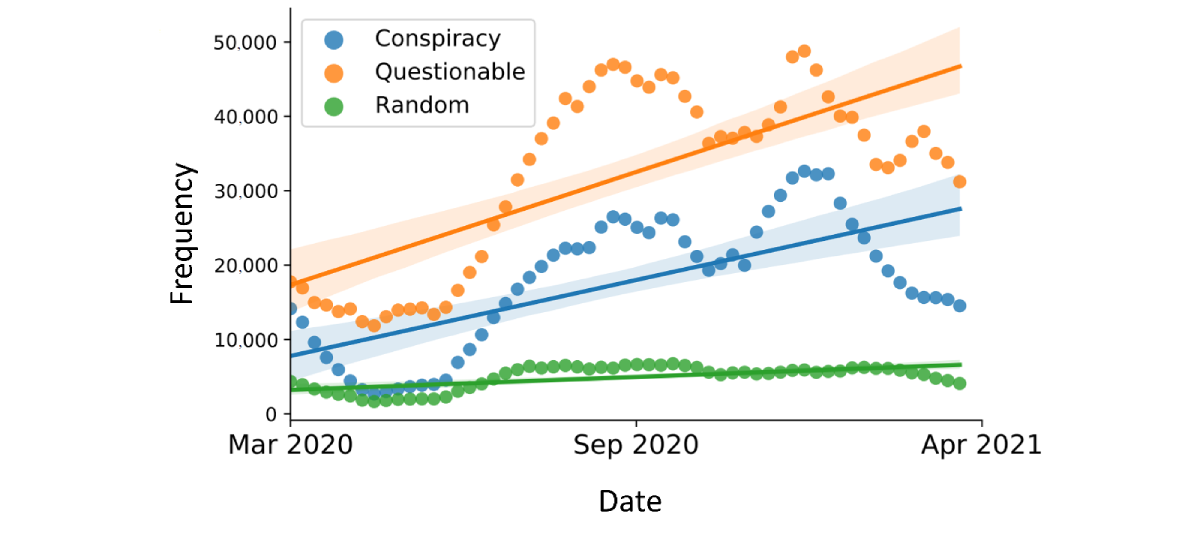
Volume of unreliable information on Twitter over time. Total number of times the news from various groups of sources were shared. The points represent the values aggregated weekly, plotted as a 7-week moving average. The lines reflect the linear trends, and the shaded areas are the 95% CIs.

The prevalence of information shared from unreliable sources is known to be high on Twitter and can reach up to 40% depending on the classification criteria [72]. In our analysis, we did not focus solely on quantifying the amount of obviously false claims, but rather focused on the prevalence of information coming from domains known to share news with questionable factualness. To obtain a more complete picture of the spread of unreliable information related to COVID-19, we performed a longitudinal analysis by quantifying the temporal trends in the volume of information shared from *conspiracy*, *questionable*, and *random* sources (see the Methods section). Figure 6 illustrates the volume of content shared from *conspiracy*, *questionable*, and *random* sources over time, plotted using a 7-week moving average. By observing the absolute trends, we can conclude that the volume of content coming from unreliable sources is growing faster than the random baseline. We modeled the change in the amount of content over time and observed a statistically significant increase in the volume of content from both groups of tracked sources, with *β_C_*=4.4740 and *β_Q_*=5.6964 representing the linear coefficients for *conspiracy* and *questionable* sources, respectively, and with *P<.*001 for both categories of sources.

We observed a large and significant increase in the amount of content from *conspiracy* and *questionable* sources. Every day, on average, we observed an increase in the amount of *conspiratorial* URLs of 4.47 and *questionable* URLs of 5.69, when corrected for the average increase of *random* content on the platform. This trend should not be overlooked, as it shows that unreliable information is on the rise despite the known efforts by Twitter to curb the spread of misinformation.

#### Audiences and Communities Sharing Unreliable Information

**Figure 7:**
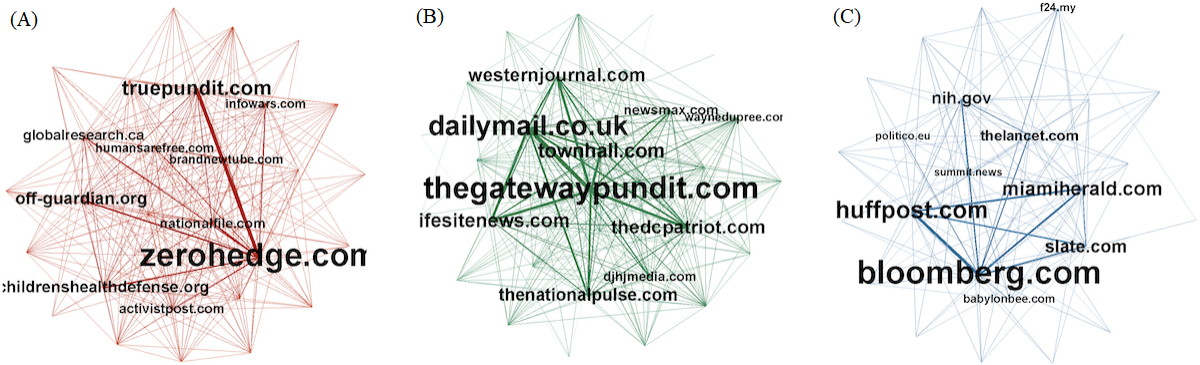
The network of audiences sharing information from various types of sources: (A) conspiracy sources, red; (B) questionable sources, green; and (C) random sources, blue. The nodes are domains that serve as the source of information. A link is drawn between the nodes if the corresponding domains have been shared by the same account. The weight of the link quantifies the number of users sharing the information from 2 domains. Each network consists of 30 nodes, randomly selected from the corresponding group of sources.

We considered the audiences and communities formed by users sharing from unreliable resources. We used the 3 domain sharing networks constructed for each group of domains: *conspiracy*, *questionable*, and *random* domain sources. The link between 2 domains was equal to the number of users who shared content from both domains. Each network comprised 250 nodes (domains). In Figure 7, only a sample of each network with 30 nodes is illustrated. From visual inspection, the networks of unreliable URLs clearly appeared to be more densely connected, suggesting greater levels of information sharing between the users and a tighter community structure.

The average clustering coefficients [21] of the *questionable* sources network and *conspiracy* sources network were 66.2 times and 27.4 times higher, respectively, than the average clustering coefficient of the *random* sources network (see Table 4 for network density measures). This is a strong indication that the connections between the URLs belonging to both groups of unreliable sources are more tightly grouped than the average set of URLs. Similarly, the average link weights of both unreliable sources’ networks are orders of magnitude higher than the average link weight of the random source’s network. The average link weights, which quantify the average number of users sharing the information from the same pair of domains, indicate that the audience sharing content from unreliable sources clusters more tightly together than the audience sharing random sources on Twitter.

**Table 4:** Some measures quantifying the connectivity of the URL networks.

Variable	Questionable sources	Conspiracy sources	Random sources
Average clustering coefficient	0.0004	0.00016	0.000006
Relative^a^ average clustering coefficient	66.21	27.43	1
Average link weight	4.69	1.36	0.01
Relative^a^ average link weight	346.69	103.15	1

^a^Relative to the network of random sources.

## Discussion

### Understanding COVID-19 Narratives on Twitter

In this paper, we provide a comprehensive overview of public COVID-19 discourse on Twitter by analyzing 1.4 billion COVID-19–related tweets that spanned the course of over a year. We make several important contributions in this work.

First, using temporal clustering of bigrams, we report 11 major topics of discussion. Aside from 1 topic with general COVID-related phrases that had sustained interest throughout our study period, the rest of the 10 topics were bursty and closely aligned with the progression of current events. We observed 2 types of topics. The first type included political topics that arise due to congregation, such as the protests that occurred in the wake of George Floyd’s death, Trump’s rallies, and India’s national exams. The second type encompassed news events that generated significant online traction, such as Trump testing positive, vaccine updates, and the relief bill. This demonstrates that observing Twitter usage is a valid way to monitor public sentiment and important events as they unfold in the real world.

We then identified misinformation narratives by analyzing latent topics detected from tweets that shared domains that have been identified as unreliable media sources. We found that the following 3 prominent misinformation narratives emerged: hydroxychloroquine and alternative medicines, US officials and governing agencies, and COVID-19 prevention practices. Each of these narratives experienced surges in mentions and engagement, the majority of which occurred in tandem with and in response to real-world events occurring at the same time.

We also characterized misinformation adoption by analyzing the retweet social network structures of users who had retweeted at least one tweet that contained a domain classified as unreliable by MBFC. We found that there exists an alignment between the misinformation topic a user tends to engage in and that user’s political party. A large portion of the left-leaning userbase engaged specifically in COVID-19 prevention misinformation. The right-leaning userbase discussed COVID-19 prevention in the context of alternative medicines (such as hydroxychloroquine), and US officials and governing authorities. Interestingly, we observed a fracture in the right-leaning user base. Some users primarily discussed only 2 of the identified narratives (COVID-19 prevention and US officials), while others engaged with tweets surrounding all 3 narratives.

Lastly, and of great concern, we found that engagement with unreliable sources is increasing at a faster rate compared to engagement with our baseline of random sources. Our results show that, in the space of public health messaging on social media platforms, there is still significant work that needs to be done in order to combat misinformation. Although social media platforms are making efforts to stem the flow of misinformation and raise awareness of its presence, the dangers of misinformation, particularly surrounding public health, are increasingly apparent. In our network, there are dense and highly connected communities that form around unreliable sources (so-called misinformation bubbles [73]), which can serve to further promulgate health misinformation online.

### Implications

Our study highlights how social media platforms can help us to shed light on the issue and consequences of misinfodemics, particularly during an unforeseen global health crisis. Social media platforms, such as Twitter, currently employ various tactics to counter misinformation, including the use of automated misinformation tags to raise awareness and partnerships with third-party fact checkers. Our research suggests that, while efforts are being made to mitigate misinformation, misinformation continues to be a mainstay on Twitter and is still growing in prevalence in the narratives we detected on online social platforms. We can also continue to understand the kinds of communities that form around sharing unreliable sources. In particular, we found that misinformation echo chambers exist within the COVID-19 misinfodemic landscape, and that the major echo chambers align with users’ political affiliations (as determined by the political lean of the sources they engage with). This has significant implications for how we can use unreliable domain usage to not only identify more communities that are susceptible to misinformation, but also funnel resources and develop strategies to combat misinformation flow in these communities.

### Limitations

While our study leverages a large tweet data set, there are still several limitations that need to be considered when interpreting the results of our study. First, when collecting data through Twitter’s free API, we were only able to collect 1% of all tweets in real time. Even with this limitation, we were able to collect several million tweets each day. We also only conducted our study on Twitter, which has been found to be used in the United States by a more liberal and left-leaning audience [74].

Due to the ever-evolving nature of misinformation, it is difficult to accurately judge and tag individual stories on Twitter as being misinformation or not. Thus, we used MBFC’s list of unreliable domains and the domains a user decides to share as a proxy for misinformation and engagement with a known unreliable source. This, however, does not necessarily mean that every URL shared from these domains has misinformation.

We did not focus on delineating *social bots* from human users in our analysis [75]. The term *social bot* generally refers to an account that is automated through software, and detecting and characterizing bot behavior is an active research area on its own [76]. Bots are incredibly salient to the misinfodemics conversation and have been found playing roles in the perpetuation of misinformation on social networks [75,77-79]. However, this study focused on the content and veracity of narratives shared on Twitter, and we hope to explore automated manipulation in the context of infodemics in future expansions of this work.

### Conclusion

In this paper, we analyzed over 1 billion tweets posted during the COVID-19 pandemic and about the pandemic, spanning the course of over a year. We described the major topics of discussion that occurred over the broader COVID-19 Twitter discourse and identified the primary misinformation narratives that permeated the Twittersphere. We demonstrated that there are distinct misinformation echo chambers that form around specific topics and narratives, and that these echo chambers are also political echo chambers. This suggests that these echo chambers are driven by not only misinformation narratives, but also political alignment. Finally, we brought awareness to the increasing presence and consumption of unreliable content on Twitter, despite the current efforts being made to mitigate misinformation spread.

The COVID-19 pandemic and subsequent lockdowns around the world forced much of our forms of communication online, creating an environment where misinformation could more easily target a wider audience. We hope that our work will provide valuable insights into which communities are more susceptible to misinformation and contribute to laying the groundwork for other researchers in the field of misinfodemics.

## Abbreviations

API: application programming interface
CDC: Centers for Disease Control and Prevention
FDA: Food and Drug Administration
LDA: latent Dirichlet allocation
MBFC: Media Bias/Fact Check
WHO: World Health Organization
